# Epidemiology and impact of a multifaceted approach in controlling central venous catheter associated blood stream infections outside the intensive care unit

**DOI:** 10.1186/1471-2334-13-445

**Published:** 2013-09-24

**Authors:** José Francisco García-Rodríguez, Hortensia Álvarez-Díaz, Laura Vilariño-Maneiro, María Virginia Lorenzo-García, Ana Cantón-Blanco, Patricia Ordoñez-Barrosa, Ana Isabel Mariño-Callejo, Pascual Sesma-Sánchez

**Affiliations:** 1 Infectious Diseases Unit, Internal Medicine Ward, Health Area of Ferrol, SERGAS, La Coruña, Spain; 2 Internal Medicine Ward, Health Area of Ferrol, SERGAS, La Coruña, Spain; 3 Preventive Medicine Ward, Health Area of Ferrol, SERGAS, La Coruña, Spain; 4 Endocrinology Ward, Health Area of Ferrol, SERGAS, La Coruña, Spain; 5 Microbiology Ward, Health Area of Ferrol, SERGAS, La Coruña, Spain; 6 C/ San Amaro 10-12, 6° Derecha, 15403, Ferrol, La Coruña, Spain

**Keywords:** Catheter, Bloodstreams, Infection control

## Abstract

**Background:**

Outside ICUs, CVC-ABSIs epidemiology and the results of strategies for their prevention are not well known. The aim of this study was to investigate the epidemiology and the impact of a multifaceted “bundle” approach in controlling CVC-ABSIs outside ICU.

**Methods:**

From 1991 we performed prevalence studies of device and parenteral nutrition use, and prospective surveillance of all episodes of CVC-ABSIs in a 350-bed teaching hospital. CVC-ABSIs incidence/1,000 inpatient-days was calculated. An estimated CVC-ABSIs incidence/1,000 catheter-days was calculated based on the prevalence rates of catheter use and the total number of inpatient-days in each year. On november 2008, an education programme was instituted for care of catheter lines: reinforcing instructions in aseptic insertion technique, after care and hand-washing; in order to assess the adherence to these measures the quantity of alcohol-based hand-rub consumption/1,000 patient-days was quoted in litres. From January 2009, a checklist intervention for CVC insertion in ICU was started: hand hygiene, using full barrier precautions, cleaning the skin with alcoholic chlorhexidine, avoiding femoral access and removing unnecessary catheters. Compliance with the central line insertion checklist was measured by real-time audits and was achieved in 80% of cases.

**Results:**

Prevalence of use of CVC and parenteral nutrition was similar throughout the study. We followed-up 309 CVC-ABSIs cases. Estimated CVC-ABSIs rate progressively increased to 15.1/1,000 catheter-days in 2008 (0.36/1,000 inpatient-days). After the intervention, the alcohol-based hand-rub consumption increased slightly and estimated CVC-ABSIs rate fell to 10.1 /1,000 catheter-days in last three years (0.19/1,000 inpatient-days), showing a 32.9% decrease. The infection rates achieved were lower in Internal Medicine wards: decreased from 14.1/1,000 catheter-days (0.17/patient-days) in 2008 to 5.2/1,000 catheter-days (0.05/1,000 inpatient-days) in last three years, showing a 63.1% decrease. In 2009, the estimated CVC-ABSIs incidence rate was significantly lower in the Internal Medicine ward compared to the Surgery ward: rate ratio (RR) = 0.14, 95%CI: 0.03-0.60), and within the Internal Medicine ward, the estimated CVC-ABSIs incidence rate was significantly lower in 2009 compared to 2008 (RR = 0.20, 95%CI: 0.04-0.91).

**Conclusion:**

The rate of CVC-ABSIs increased outside-ICU, and the implementation of multifaceted infection control programme decreased their clinical impact.

## Background

Catheter-associated bloodstream infections are among the most frequent healthcare-associated infections and have been associated with higher cost, crude mortality rates, and number of inpatient days.

The true incidence of catheter-associated bloodstream infections is not known and resulting mortality is not clear [1]. In 2002 alone, an estimated 250,000 health care–onset catheter-associated bloodstream infections occurred in the United States, resulting in 30,000 deaths.

A complete description of prevention practices is available [2] and the results from several collaborative studies have demonstrated a roughly 70% reduction in catheter-associated bloodstream infection rates in intensive care units (ICUs) by increasing adherence to recommended best practices for the insertion of Central Venous Catheters (CVC) [3]. In 2009 the estimated number of ICU CVC-ABSIs in the United States was 18,000, a 58% reduction compared to 2001 [1,4].

To date, most prevention efforts have focused on ICUs basically due to better nurse and physician to patient staffing ratios and the higher risk populations. The validity of these results outside of ICUs is not well known. Types and use of vascular catheters, site and duration of catheterization, average patient stay, amount of antibiotic treatment given, and after care, appear to vary between ICUs and general wards, as well as unjustified use and inadequate dressing of catheters that might be more common outside ICUs [5].

For non-ICU patients, catheters are used in a smaller percentage of cases and the device utilisation ratio is lower than for ICU patients. However, 87.5% of inpatient-days occurred in an outside-ICU setting and the absolute number of catheters used was higher for non-ICU than for ICU patients. Therefore the total burden of catheter use might be higher outside ICUs and in 2009 there were an estimated 23,000 catheter-associated bloodstream infections in the United States in outside-ICU inpatient care [1,6].

Given the low infection rates that have been achieved in ICUs when recommended prevention methods have been instituted, a better understanding of the relative effectiveness and wider applicability of CVC-ABSI prevention interventions is required [7].

The aim of this study was to investigate the epidemiology and the impact of a multifaceted “bundle” approach in controlling CVC-ABSIs outside ICU.

## Methods

### Setting

The study was conduced in a 350-bed teaching hospital from 1991 to 2011 with the approval of ethics committee of Hospital A. Marcide. Throughout the time of the study the number of hospital beds was similar, with some redistribution when new hospitalisation units were opened, the number of hospital admissions increased slightly (mean 11,078 admissions per year), the mean length of hospital stay decreased from 10.3 days in 1991 to 8.74 days in 2011 and the inpatient-days per year did not change significantly. The number of admissions and inpatient-days were lower during July-September months.

Patient samples were taken as part of standard care. The hospital provides healthcare for an area with 215,000 inhabitants and has one ICU with 10 beds. An Oncology ward was opened in July 2006, in March 2008 a Nephrology ward with a programme of haemodialysis for acute patients was started, and dispensers for alcohol-based handrubs were installed in every room. In our hospital we do not have a dedicated infection control nurse.

### Study design and current practices

From 1991 hospital preventionist staff performed two or three 1-day prevalence studies per year for all beds of the hospital, during the months of March, May, and October, in 1993 another prevalence study was performed in July. According to a protocol, the following data were collected: date, sex, age, admission ward, inpatient days, type of intravascular device (peripheral or central), parenteral nutrition, surgical operation and severity of underlying diseases (McCabe and Jackson classification) [8].

In addition, from 1991 a single medical infectious diseases specialist performed prospective active surveillance of all episodes of bloodstream infections and performed an assessment of patients to determine the place of acquisition and origin of bacteremia. The bacteremias caused by an intravascular catheter associated infection were selected for the study. For each episode of bacteremia, we collected the following data: date, admission ward, sex, age, type of intravascular device (peripheral or central), parenteral nutrition, surgical operation, bacteria or fungi, severity of underlying diseases and clinical progress.

### Definitions

Nosocomial bacteremia and CVC-ABSIs were defined using the Centre for Disease Control criteria [9]. A bacteremia was considered nosocomial if it was detected at least 48 hours after admission and also if the bacteremia was associated with the use of intravascular catheters in an out of hospital setting and detected in 48 hours after admission. CVC-ABSI must meet one of the following criteria: 1. The patient must have a recognised pathogen cultured from one or more blood cultures, with the organism cultured from blood not related to an infection at another site; 2. The patient must have at least one of the following signs or symptoms: pyrexia (> 38°C), chills or hypotension; signs and symptoms of infection and positive laboratory results not related to an infection at another site, and common skin contaminant (i.e.diphtheroids, [*Corynebacterium spp*.], *Bacillus spp*. [not *B. anthracis*], *Propionibacterium spp*., *coagulase-negative Staphylococci* [including *S. epidermidis*], *Streptococcus viridans*, *Aerococcus spp*. and *Micrococcus spp*.) cultured from two or more cultures drawn on separate occasions.

The identification of blood isolates was performed according to Clinical Laboratory Standard International. Procurement of blood cultures and intravascular tip cultures was determined, as clinically indicated, by the primary medical team. Patients were eligible to have more than one infectious event if the episode was determined to be unrelated to the previous episode.

A CVC-ABSI was considered to be related to a specific ward if it was detected at least 48 hours after admission to or less than 48 hours after discharge from the unit. CVCs are defined as intravenous catheters that end at or near the heart, or in a great vessel close to the heart (eg, subclavian vein, internal jugular vein, femoral vein). Peripherally inserted catheters that enter the superior vena cava and totally implanted catheters (Port-a-Cath) are also considered CVCs. CVC-ABSIs attributable mortality was considered when the patient died within 7 days of catheter-associated bloodstream infection and there was no other clear explanation for the outcome.

### Catheter placement and care

Throughout the time of the study CVCs were placed in the subclavian and jugular vein in descending order of frequency. Aqueous povidone-iodine solution was used as a skin antiseptic for CVCs inserted by physicians outside ICU and for all catheters after care performed by ward nurses outside ICU. Catheter care in ICU was performed with alcoholic chlorhexidine solution. Chlorhexidine-impregnated sponge dressings, tunnelled or impregnated catheters were not used.

### Interventions

In 1996 a hospital infection prevention and control guide was prepared and distributed to ward supervisors to present its content to nursing and medical staff. CVC-ABSIs increased in the period to 2008, with a relatively large rise between 2006 to 2008, and in November 2008 an enhanced, multifaceted catheter-associated bloodstream infection control programme was instituted:

1. Course on nosocomial infection prevention and control was given to nursing and medical staff, with voluntary attendance of 30 people; prevention measures on aseptic insertion technique of catheter line and after care, changing gauze dressing every 72–96 hours or more frequently if the dressing was wet, loose or when redness was observed at the site, hand washing and appropriate use of gloves were reinforced. In order to assess the adherence to these measures the quantity of alcohol-based hand-rub consumption was quoted in litres per 1,000 patient-days.

2. From January 2009, a hospital protocol for parenteral nutrition treatment was implemented, introducing the need to provide parenteral nutrition through central line catheters with special supervision.

3. From January 2009 to december 2011 a national program called “bacteremia zero” was followed in the ICU-service with the implementation of 5 measures relating to the insertion and maintenance of CVCs and using a checklist for the insertion [10]: a. hand hygiene, b. using full barrier precautions, c. cleaning the skin with a 2% chlorhexidine gluconate with 70% isopropyl alcohol solution; d. avoiding femoral access and e. removing unnecessary catheters. Compliance with the central line insertion checklist was measured by real-time audits and was achieved in 80% of cases.

4. The educational programme and protocol for parenteral nutrition were also displayed on the hospital intranet from the beginning of 2009.

No other infection-reducing interventions were initiated during the study period.

### Outcome measures

To assess the efficacy of our interventions, we recorded (before and after the intervention) the epidemiology and clinical features of CVC-ABSIs in non-ICU inpatients. CVC-ABSIs incidence/1,000 inpatient-days was calculated. CVC-ABSIs incidence/1,000 catheter-days for each unit-year was estimated based on data of the prevalence of catheter use and the total number of patient-days in each year (obtained from administrative data). Data from prevalence studies were used to calculate a sample device utilization ratio (the number of line days divided by the number of patient-days in the sample). The sample device utilization ratio was multiplied by the total number of inpatients-days per unit-year to yield the estimated line-days [11]. CVC-ASBIs incidence is expressed as the number of CVC-ABSI cases divided by the number of estimated CVC-days, standardised as a rate per 1,000 catheter-days.

### Statistical analyses

The results were analysed using the commercially available SPSS software, version 14.0. A descriptive and comparative study of the variables was performed. Continuous variables were analysed using Student’s t-test and categorical data were analysed using the chi-square or Fisher’s exact test, as appropriate. Associations between the variables were expressed as unadjusted odds ratios (ORs) and 95% confidence interval (CIs). The chi-square for trend was used to compare evolution of proportions. CVC-ABSI rate with a 95% confident interval was calculated as Poisson event rates, and compared by testing for homogeneity of rates. Statistical significance was set at 2-sided p < 0.05.

## Results

### Prevalence studies

In the prevalence studies, 10,114 patients were surveyed, 9,899 (97.9%) were non-ICU inpatients. Over time the mean age of patients and severity of the underlying rapidly fatal illness increased. In this way, out of 9,899 non-ICU inpatients: 1,968 patients were surveyed between 1991and 1995: mean age 56.6 ± 20.6 years and underlying rapidly fatal illness 8.1%; 1,995 patients were surveyed between 1996 and 2000: mean age 58.3 ± 19.8 years and underlying rapidly fatal illness 9.3%; 2,512 patients were surveyed between 2001 and 2005: mean age 61.5 ± 19.1 years and underlying rapidly fatal illness 7.3%; and 3,424 patients were surveyed in last 6 years: mean age 63.1± 19.6 years and underlying rapidly fatal illness 13.7%. The use of CVC was associated with underlying rapidly fatal illness compared to ultimately and non fatal illness (OR 3.3, 95%CI: 2.4-4.6, p<0.001) and with higher mean age of patients (72.3 ± 15.3 vs 59.3± 19.8 years, p<0.001).

Female gender prevalence, prevalence of surgical operation, prevalence of CVC use and parenteral nutrition utilisation were not modified over time.

The estimated number of catheter-days retrieved from the prevalence studies varied significantly over time, ranged from 969 in 1991 up to 2,587 in 2008 and then fell until 1,285 catheter-days in 2011, p<0.05. These fluctuations were related to changes in the number of admissions, surgical activity and temporary reduction in the number of beds due to restructuring works of some hospital wards.

In the summer of 1993, prevalence of CVC use and parenteral nutrition utilisation were similar to the rest of that year (in summer, prevalence use of CVC 3,1% vs 2.3%; parenteral nutrition use: 2.3% vs 1.5%, no significant difference).

### Bacteremia surveillance

The number of blood cultures performed/1,000 inpatient-days increased from 13.8 in the year 1998 to 29.2 in 2011 (administrative data about the total number of blood cultures performed during previous years are not available).

One thousand three hundred and eighteen cases of nosocomial bloodstream infections were followed, 607 (46%) of them were catheter line-ABSIs. Among the 607 episodes, 530 (87.3%) were in non-ICU patients and of 530, 309 (58.3%) were CVC-ABSIs, in 260 patients. Two hundred and twenty-four had one episode of CVC-ABSI and 36 patients had more than one episode: 25 two episodes, 9 three episodes and 2 four episodes.

Microbiological Characteristics: 251 (90%) of the 279 cases of monomicrobial CVC-ABSIs were caused by gram-positive bacteria: 210 isolates (75.3%) were coagulase-negative staphylococci, 32 (11.5%) were *Staphylococcus aureus* [3 (1.1%) Methicillin resistant], 2 (0.7%) were *Enterococcus* species, and 7 (2.5%) were other gram-positive organisms. Gram-negative bacteria caused 16 (5.7%) of the monomicrobial CVC-ABSIs: 4 isolates (1.4%) were *Escherichia coli*, 3 (1.1%) *Pseudomonas aeruginosa*, 3 (1.1%) *Klebsiella pneumoniae*, 2 (0.7%) *Serratia marcescens*, 1 (0.3%) was *Proteus mirabilis*, 1 (0.3%) *Klebsiella oxytoca,* 1 (0.3%) *Morganella morganii* and 1 (0.3%) was *Aeromonas spp*, and there were not ESBL-producing strains among enterobacteriaceae isolates. Furthermore, 9 (3.2%) monomicrobial CVC-ABSIs were caused by *Candida albicans*, and 3 (1.1%) were caused by *Candida* species other than *C. albicans*. Finally, 30 CVC-ABSIs (9.7%) were polymicrobial. Cultures were not obtained from all CVCs after removal and 229 of 309 (74.1%) were confirmed by culture of the catheter tip.

The distribution of 309 CVC-ABSIs by admission ward was: General Surgery 182, Internal Medicine 63, Haematology 23, Urology 20, Nephrology 8, others 13.

CVCs were inserted by anaesthetists, surgeons, ICU physicians, internists and nephrologists in 55.3%, 20.7%, 19.1%, 2.6% and 2.3% of cases, respectively, and were placed in subclavian (73.6%), jugular (22.9%) and femoral veins (2.6%). Thirty one (10%) of CVC-ABSIs were from totally implanted ports.

Among 309 cases of CVC-ABSIs, 117 (37.9%) were females and the mean age of the patients was 60.6 ± 15.4 years (rank 16–90). CVC-ABSIs were associated with parenteral nutrition in 237 (76.7%) cases, surgical intervention in 170 (55%), cancer in 152 (49.2%) and diabetes mellitus in 59 (19.1%). Among 309, 56 (18.1%) cases had underlying rapidly fatal illness and 14 cases died due to a bacteremia episode. CVC-ABSIs attributable mortality was related to diabetes mellitus (42.9%; OR: 3.4, 95%CI: 1.14-10.3) and to infection by *Staphylococcus aureus* (42.9%; OR: 6.39, 95%CI: 2.1-19.6).

The distribution per months showed higher incidence during July-September (0.25/1,000 inpatient-days) than in the rest of the year (0.17/1,000 inpatient-days), and the increase in the number of cases in summer was evident throughout all the years of the study. CVC-ABSIs attributable mortality was higher during the summer months (8.4% vs 2.8%; OR: 3.2, 95%CI: 1.1-9.46), p<0.05.

### Outcome of bundles

**Figure 1:**
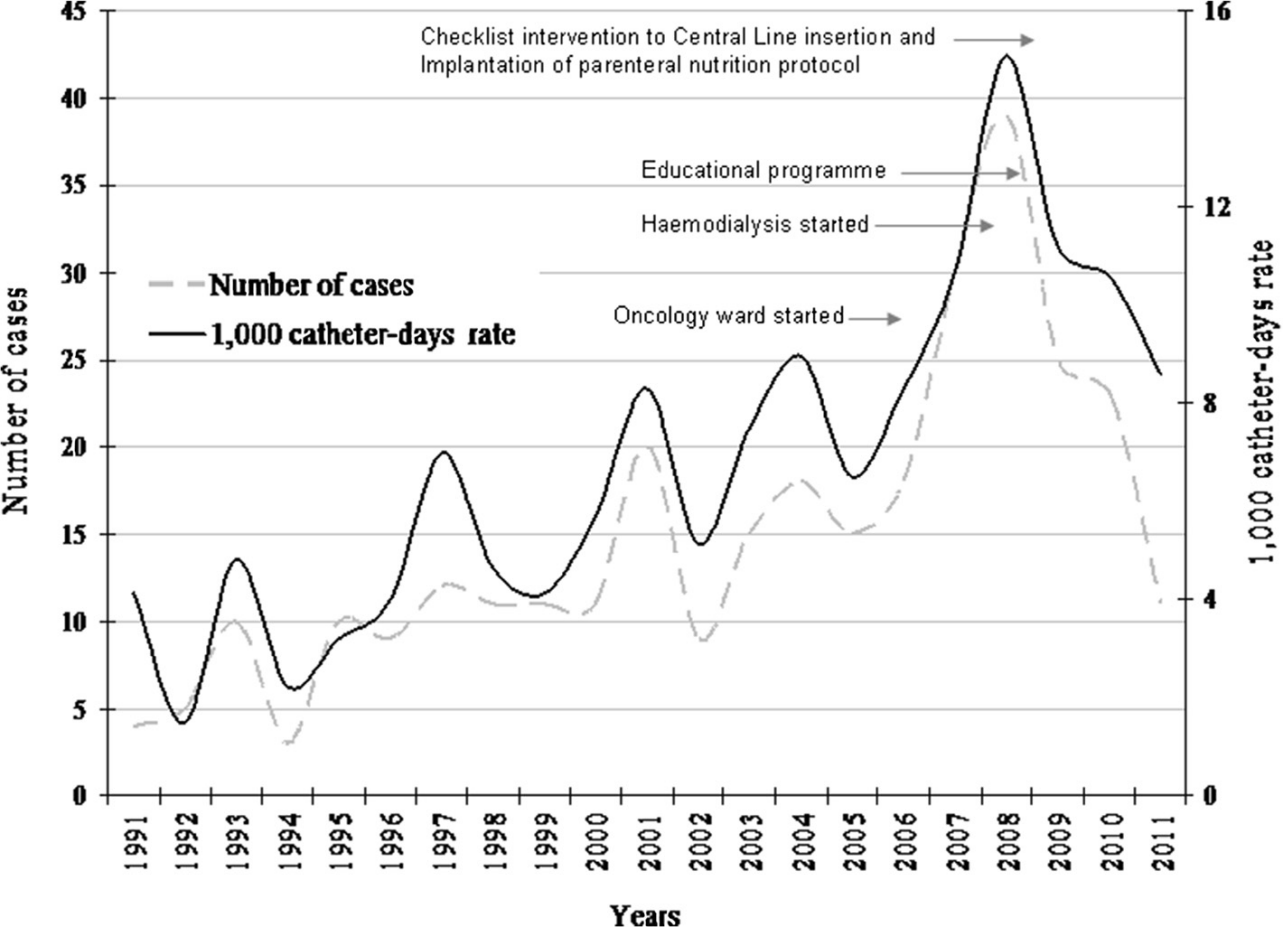
Evolution of CVC-ABSIs rate outside-ICU, years 1991–2011.

Estimated CVC-ABSIs rate progressively increased to a maximum of 15.1/1,000 catheter-days (0.36/1,000 inpatient-days) (Figure 1). After the intervention, estimated CVC-ABIs rate fell to 10.1 /1,000 catheter-days in last three years (0.19/1,000 inpatient-days), showing a 32.9% decrease (Table 1). The estimated CVC-ABSIs incidence/1,000 catheter-days tended to be lower in Internal Medicine than in Surgical wards, and the decrease achieved was higher in Internal Medicine: from 14.1/1,000 catheter-days (0.17/1,000 inpatient-days) in 2008 to 5.2/1,000 catheter-days (0.05/1,000 inpatient-days) in last three years, showing 63.1% decreased. In 2009, the estimated CVC-ABSIs incidence rate was significantly lower in the Internal Medicine ward (2.8; 95%CI: 0.6-10) compared to the Surgery ward (20.2; 95%CI: 12–33), p = 0.0013; rate ratio (RR) = 0.14, 95%CI: 0.03-0.60. Within the Internal Medicine ward the estimated CVC-ABSIs incidence rate was significantly lower in 2009 (2.8; 95%CI: 0.6-10) compared to 2008 (14.1; 95%CI: 6.5-27), p = 0.026; RR = 0.20, 95%CI: 0.04-0.91).

**Table 1:** Epidemiology and the impact of multifaceted bundle approach in controlling CVC-ABSIs in non-ICU inpatients, year before and three years after the intervention

	**Years**				**p**
	**2008**	**2009**	**2010**	**2011**	
Prevalence use of central line	15/632 (2.4%)	13/616 (2.1%)	11/573 (1.92%)	8/649 (1.2%)	0.50
Surgery ward#	5/120 (4.2%)	4/115 (3.5%)	5/133 (3.8%)	5/118* (4.2%)	0.99
Internal medicine ward#	5/266 (1.9%)	6/290 (2.1%)	3/276 (1.1%)	3/334* (0.9%)	0.57
Estimated number of catheter-days	2,587	2,233	2,176	1,285	<0.05
Prevalence use of parenteral nutrition by central line	8/15 (53.3%)	5/13 (38.5%)	5/11 (45.5%)	6/8 (75%)	0.83
Surgery ward#	4/5 (80%)	3/4 (75%)	4/5 (80%)	5/5 (100%)	0.99
Internal medicine ward#	2/5 (40%)	0/6 (0%)	0/3 (0%)	1/3 (33.3%)	0.41
Mean age ± SD	60.2 ± 20.9	63.2 ± 19.5	64.9 ± 19.9	65.9 ± 18.5	<0.05
Mean length of stay (days) in surgery ward	10.93	10.61	10.98	10.37	0.97
Mean length of stay (days) in internal medicine ward	9.58	9.36	9.33	9.17	0.95
CVC-ABSIs surveillance					
No. of blood cultures performed/1,000 inpatient-days	23.3	27.1	29.5	29.2	
No. of CVC-ABSIs / No. of blood cultures performed	39/2880	25/3275	23/3483	11/3422	<0.001
Positive catheter tip culture	27/39 (69.2%)	18/25 (72%)	14/23 (60.9%)	11/11 (100%)	0.83
No. CVC-ABSIs /1,000 catheter-days	15.1	11.2	10.6	8.6	
Surgery ward	18.9	20.2*	14.1	8.9	
Internal medicine ward	14.1	2.8*	8.7	4	
Alcohol-based handrubs consumption. L/1,000 inpatient-days	2.34	6.07	3.5	5,2	
Surgery ward	1.1	5.6	3.76	4.6	
Internal medicine ward	2.2	6.78	4.64	4.97	

*Differences between Surgery and Internal Medicine ward were significant in this year and estimated CVC-ABSIs incidence rate in Internal Medicine was significantly lower in 2009 compared to 2008, p<0.05. #Differences in prevalence use of CVC and parenteral nutrition between Surgery and Internal Medicine wards are significant when comparing the sum total of the years, p<0.05. Statistical analyses: Chi-square for trend. Poisson model to calculate and compare rates.

From 2008 to 2009–2011, the CVC device utilization ratio (0.39 in 2008 vs 0.14 in 2011, p<0.001) and estimated number of catheter-days per year decreased and the rate of alcohol-based handrub consumption increased slightly (no statistically significant). There was no significant difference between the years in number of line days (10±4.4 days in 2008 vs 12.3±8.2 in 2011) and in other variables analysed.

## Discussion

Our results indicate that CVC-ABSIs rates are increasing and more than 85% of CVC-ABSIs occur in the non-ICU setting, accounting for 33% of largely preventable CVC-ABSIs with the implementation of a multifaceted infection control programme. The decrease achieved in CVC-ABSIs rates in the first year of the intervention was higher and significant in medical wards, adjusted per catheter and per patient-days.

The prevention and control of CVC-ABSIs has rarely been investigated outside the ICU setting, and to our knowledge, there is only one study conducted at two small community hospitals demonstrating 57% CVC infection rate reduction in non-ICU CVC-ABSIs, with the use of antibiotic impregnated catheters for some patients [12]. In this series the use of CVC, as we also reported, was higher in non-ICU setting, and considering that the vast majority of all hospitals are community hospitals with fewer than 200 beds, these results reinforce the necessity and importance of restoring measures of prevention and control in non-ICU inpatient [6,13].

The results in non-ICU floors were lower than 65%-70% decrease for ICUs. However, the results are not comparable because of differing patient characteristics and nurse and physician to patient staffing ratios [14]. The introduction of the bundle from January 2009 did not result in the levels falling below those achieved before 2006–2008, possibly because in those years the majority of oncology patients and patients included in haemodialysis programme were not admitted to our hospital.

In our study, the outside-ICU estimated CVC-ABSIs/1,000 catheter-days incidence is higher than 1–2.9/1,000 catheter-days of laboratory-confirmed central line associated bacteremia rates from data reported by hospitals participating in the National Healthcare Safety Network (NHSN) between January and December 2009 [15]. Our incidence is also higher than 4.3/1,000 inserted CVC-days from Internal Medicine and Surgical wards in German hospitals [16], higher than 4.4 in an General Medical service [13], higher than 6.6 in burn patients [17] and than 0.87 in patients with solid tumours receiving chemotherapy in an ambulatory setting [18]. Our greater incidence could be due to: our inclusion of all cases of CVC-ABSIs clinical sepsis, we have less dedicated infection control personnel than in other centers, the existence of best results publication bias and the method we used to calculate the incidence. Collecting information on central line-days is labor intensive and the rate per 1,000-catheter-days in our study was based on an estimation of catheter use [11]. The size of the prevalence study sample and the number of cases of CVC-ASBIs were small and it is possible that the incidence rates are not very reliable and could be overvalued. However, having used the same method of estimation over time should not subtract solidity to the evolution of the incidence before and after bundles, as is proven achieving similar results when analyzing the evolution of CVC-ABSIs rate per patient days.

The strengths of our study are that surveillance was performed by staff with formal infection control training and who have years experience working in infection control [19], that the study provides hospital-wide surveillance information and reports secular trends over two decades. Our results indicate that there are temporal variations in the incidence and differences between surgical and medical wards. The incidence increased possibly due to an increasing use of these catheters in a progressively ageing population, with greater comorbidity and more invasive treatments, as well as due to better detection of blood stream infections.

CVC-ABSIs were associated with parenteral nutrition use and CVC-ABSIs rate was higher in surgical rather than medical wards. CVC and parenteral nutrition use was similar over time, but was greater in surgical wards and this could explain the difference in rate between admission wards [20]. On the other hand, it is necessary to consider that patients sometimes need to be transferred from one ward to another, which can make it difficult to allocate CVC-ABSIs to a specific unit, and the results of one ward could show the effect of measures of prevention implemented in another unit [13,20]. In this way, the ICU of our hospital participates from 2009 in the national program called “bacteremia zero” which was developed to reduce the CVC-ABSIs in patients with higher risk of this complication, and it is possible that the higher and significant rate reduction that was achieved in Internal Medicine could be explained in part because 65% of all patients with CVC followed there (with and without CVC-ABSI) were patients with CVC inserted by ICU physicians.

Another issue that could have contributed to different results is the difference in staff training and their motivation to participate in the prevention and control programme [16]. This seems to be reflected in the different alcohol-based hand-rub consumption and by the increased CVC-ABSIs rate in the summer. This increase of CVC-ABSIs numbers in summer, a period of time with no apparent difference in the use of CVCs than in the rest of year and with a smaller number of admissions and inpatient-days (administrative data), makes us to formulate the hypothesis that this could be explained by the temporal recruitment of nurses with less experience in the handling of the catheters to work for the hospital.

We are unable to determine whether specific interventions led to the observed decrease in CVC-ABSIs rates because several measures were started simultaneously. Although a standard control group for comparison purposes was not feasible, the fact that a progressive and largely consistent increase in CVC-ABSIs rates from 1991 to 2008 and a decrease in CVC-ABSIs rates was observed for three years after starting the intervention programme, could support a cause and effect relationship between the beginning of a programme called “bacteremia zero” and a decrease in outside ICU CVC-ABSIs rates in Internal Medicine wards. The effects of this intervention and the increase in CVC-ABSIs rates during summertime reinforce the importance of allocating only trained and competent personnel for insertion and maintenance of central intravascular catheters, and ensuring appropriate nursing staff levels in non-ICU settings [2,12].

Improving the measures of asepsis on insertion and after care of catheter, and removing the unnecessary catheters could have a very important impact [21-23], as it seems to be evidenced by a 64.1% reduction in the catheter-utilization ratio of CVC inserted in the last year. This decrease in CVC exposure could in part explain a decrease in CVC-ABSI incidence.

There are several limitations to this study. First, we did not collect data on pre and post intervention process measure compliance, and this lack of data decreased our ability to measure the full impact of the bundles. Secondly, this is an single-centre study and we have estimated incidence/1,000 catheter-days [11] because we do not know the incidence and duration of inserted catheters, which makes it difficult to generalise practice patterns to other centres, given that infection control policies may differ significantly between different hospitals. Thirdly, catheter insertion sites were not documented, therefore, infection rates attributable to a specific insertion site could not be analysed. Fourthly, cultures were not obtained from all CVCs after removal.

## Conclusions

Our results indicate that the majority of bacteremias associated with central venous catheter use occurred in non-ICU settings, one third of these catheter associated bloodstream infections could be prevented by the implementation of a multifaceted infection control programme with different results being achievable according to specialty of admission ward.

## Competing interests

The authors declare that they have no competing interest.

## Authors’ contributions

GR performed surveillance of bloodstream infections, conceived of the study, participated in its design and wrote the first draft of the manuscript. LG performed prevalence studies and contributed to the interpretation of the results and critical revisions of the manuscript. AD, VM, CB, OB, MC and SS contributed to the interpretation of the results and critical revisions of the manuscript. All authors read and approved the final manuscript.

## Pre-publication history

The pre-publication history for this paper can be accessed here:

http://www.biomedcentral.com/1471-2334/13/445/prepub
